# Up-regulation of long non-coding RNA CYTOR induced by icariin promotes the viability and inhibits the apoptosis of chondrocytes

**DOI:** 10.1186/s12906-021-03322-1

**Published:** 2021-05-26

**Authors:** Guoyou Wang, Lei Zhang, Huarui Shen, Qi Hao, Shijie Fu, Xia Liu

**Affiliations:** 1 Department of Orthopaedics, Hospital (TCM) Affiliated To Southwest Medical University, Chunhui Road No.182, Longmatan District, Luzhou, 646100 Sichuan China; 2 Department of Law, Southwest Medical University, Xianglin Road Section 1 No.1, Longmatan District, Luzhou, 646100 Sichuan China

**Keywords:** Icariin, Osteoarthritis, chondrocytes, LncRNA CYTOR

## Abstract

**Background:**

Icariin (ICAR) is the main effective component extracted from epimedium, and is reported to have the potential to treat osteoarthritis (OA). However, its pharmacological function on chondrocytes has not been fully clarified.

**Methods:**

Different doses of ICAR were used to treat chondrocyte cell lines, including CHON-001 and ATDC5. Then the expressions of different lncRNAs were measured by qRT-PCR. Interleukin-1β (IL-1β) was used to simulate the inflammatory response environment of chondrocytes. Overexpression plasmids and short hairpin RNAs of lncRNA CYTOR were used to construct gain-of-function and loss of function models. CCK-8 was conducted to determine the cell viability. Flow cytometry was used to detect the apoptosis of chondrocytes. Enzyme-linked immunosorbent assay (ELISA) was adopted to measure the contents of inflammatory factors (IL-6, IL-8, TNF-α) in the supernatant of the chondrocytes.

**Results:**

Compared with other lncRNAs, CYTOR was changed most significantly in both CHON-001 and ATDC5 cells after treatment with ICAR. ICAR promotes the viability and inhibits the apoptosis of CHON-001 and ATDC5 cells induced by IL-1β, accompanied with reduced levels of inflammatory factors. Overexpression of CYTOR facilitated the viability of chondrocytes, while repressed their apoptosis and inflammatory response. What’s more, knockdown of CYTOR reversed the protective effects of ICAR on chondrocytes.

**Conclusion:**

CYTOR was a pivotal lncRNA involved in the protective function of ICAR on chondrocytes.

## Background

Osteoarthritis (OA) is a chronic joint degenerative disease characterized by pain, activity restriction, and joint stiffness [1]. Reportedly, among the people over the age of 60, about 10% of men and 13% of women suffer from OA [2]. Non-steroidal anti-inflammatory drugs is the main drug for the treatment of OA, but they induce the side effects such as gastrointestinal discomfort, and they can’t repair the damaged cartilage [3]. In OA, different biochemical changes can affect the viability and function of chondrocytes, thereby promoting the degradation of the cartilage matrix and eventually leading to cartilage destruction [4]. Inhibiting the inflammatory injury of chondrocytes may a promising strategy to treat OA.

Icariin (ICAR) is one of the main bioactive components of *Epimedium* [5]. In recent years, especially in China, as a kind of adjuvant therapy, ICAR is applied to treat bone diseases such as osteoporosis and fracture [6]. In OA, it is reported that ICAR can inhibit lipopolysaccharide (LPS)-induced chondrocyte inflammation; in addition, it can also inhibit the caspase-1 signal pathway mediated by NLRP3 inflammasome, thus reducing LPS-induced chondrocyte pyroptosis [7]. Nonetheless, the detailed mechanism by which ICAR regulates OA pathogenesis has not been fully clarified.

Long non-coding RNA (lncRNA) is a kind of RNA with a length of more than 200 nucleotides that does not have the ability to encode proteins, and reportedly, lncRNA can regulate the proliferation, differentiation, apoptosis, and inflammatory responses of different cells, including chondrocytes [8]. Multiple lncRNAs have been linked to the pathogenesis of OA and the dysfunction of lncRNAs, such as HOTAIR, PACER, CILinc01, ZFAS1, XIST, MALAT1, RP11-249C24.10 and RP11-855A2.5, CRNDE, cytoskeleton regulator RNA (CYTOR) and so on [9]. However, it is unclear whether ICAR plays a role in treating OA by regulating these lncRNAs.

Therefore, the purpose of this study was to investigate the relationship between ICAR and lncRNA in chondrocytes. We used interleukin 1β (IL-1β) to treat chondrocyte cell lines CHON-001 and ATDC5 to establish in vitro model of OA, and then the effects of different concentrations of ICAR on the viability, apoptosis and inflammatory responses of chondrocytes were detected. It was found that ICAR significantly increased the expression of CYTOR to protect the joint cartilage in inflammatory conditions.

## Methods

### Cell culture and transfection

The human chondrocyte cell line CHON-001 and mouse chondrocyte cell line ATDC5 were purchased from the American Tissue Culture Collection (ATCC, Manassas, VA, USA). These cells were cultured in Dulbecco’s Modified Eagle’s Medium (Gibco, Grand Island, New York) supplemented with 10% fetal bovine serum (FBS) (Gibco, Grand Island, New York) and 100 U / mL penicillin and 100 μg / mL streptomycin (Hyclone, Logan, UT, USA). All cells were cultured in a humidified incubator containing 5% CO_2_ at 37 °C. IL-1β (Sigma-Aldrich, Shanghai, China) was diluted to 10 ng / ml with serum-free medium, and CHON-001 and ATDC5 cells were stimulated with 10 ng/ml IL-1β to establish OA models. Subsequently, different concentrations of ICAR (Tauto Biotech, Shanghai, China) (0, 10, 20, 30 μM) were used to treat the cells. CYTOR overexpression plasmids and CYTOR short hairpin RNA (sh-CYTOR) were constructed by GeneChem (Shanghai, China). The transfection was performed with Lipofectamine® 3000 (Invitrogen, Carlsbad, CA, USA) according to the manufacturer’s instruction. 48 h later, the cells were harvested for subsequent experiments.

### Quantitative real-time polymerase chain reaction (qRT-PCR)

TRIzol reagent (Life Technologies, Carlsbad, CA, USA) was used to extract total RNA from CHON-001 and ATDC5 cells, and the Primescript™ reverse transcription kit (TaKaRa, Shiga, Japan) was adopted to reversely transcribed the RNA into complementary DNA, and qRT-PCR was performed with SYBR Green Master Mix kit (Takara, Otsu, Japan) on ABI 7500 Real-Time PCR system (Applied Biosystems, Carlsbad, CA, USA) to analyze the relative expression levels of CYTOR. GAPDH was used to standardized the expression levels with 2^-ΔΔCt^ Methods. The primer sequences were provided by BGI (Shenzhen, China).

### Cell counting kit-8 (CCK-8) assay

CCK-8 kit (Sigma-Aldrich, St. Louis, Mo, USA) was used to detect the cell viability of the CHON-001 and ATDC5 cells. 2 × 10^3^ cells were inoculated into each well of a 96-well plate, and the cells were cultured for 24, 48 or 72 h. At each time point, 10 μL of CCK-8 kit was added into each well, and the cells were incubated at 37 °C in 5% CO2 for 1 h. Subsequently, a microplate reader (Bio-Rad Laboratories, Inc., Hercules, CA, USA) was used to detect the optical density (OD) value of the cells in each group at a wavelength of 450 nm.

### Flow cytometry analysis

ATDC5 and CHON-001 cells were harvested, and washed with PBS twice. Then Annexin V-FITC/propidium iodide (PI) detection kit (BD Pharmingen, San Jose, CA, USA) was used to analyze the apoptosis of Chondrocytes. In each group, about 5 × 10^5^ chondrocytes were resuspended with 100 μL of binding buffer. Next, 5 μL of Annexin V-FITC staining solution and 5 μL of PI staining solution were added in the binding buffer, and the cells were incubated in the dark for 15 min at room temperature. Ultimately, the apoptotic rates of chondrocytes were analyzed by a flow cytometer (Beckman Coulter, Fullerton, CA, USA).

### Enzyme-linked immunosorbent assay (ELISA)

The supernatant of CHON-001 and ATDC5 cells were collected after high-speed centrifugation. The concentrations of IL-6, IL-8, TNF-α in the supernatant was measured by ELISA method with the corresponding kit (eBioscience, San Diego, CA, USA) according to the manufacturer’s instructions.

### Statistical analysis

All experiments were repeated in triplicate. SPSS22.0 (IBM Corp., Armonk, NY, USA) was used for statistical analysis. The experimental data were expressed as mean ± standard deviation, and the difference between the two groups was compared using Student’s *t*-test. *P* < 0.05 indicated that the difference was statistically significant.

## Results

### IL-1β induced inflammatory injury to chondrocytes in vitro

**Fig. 1:**
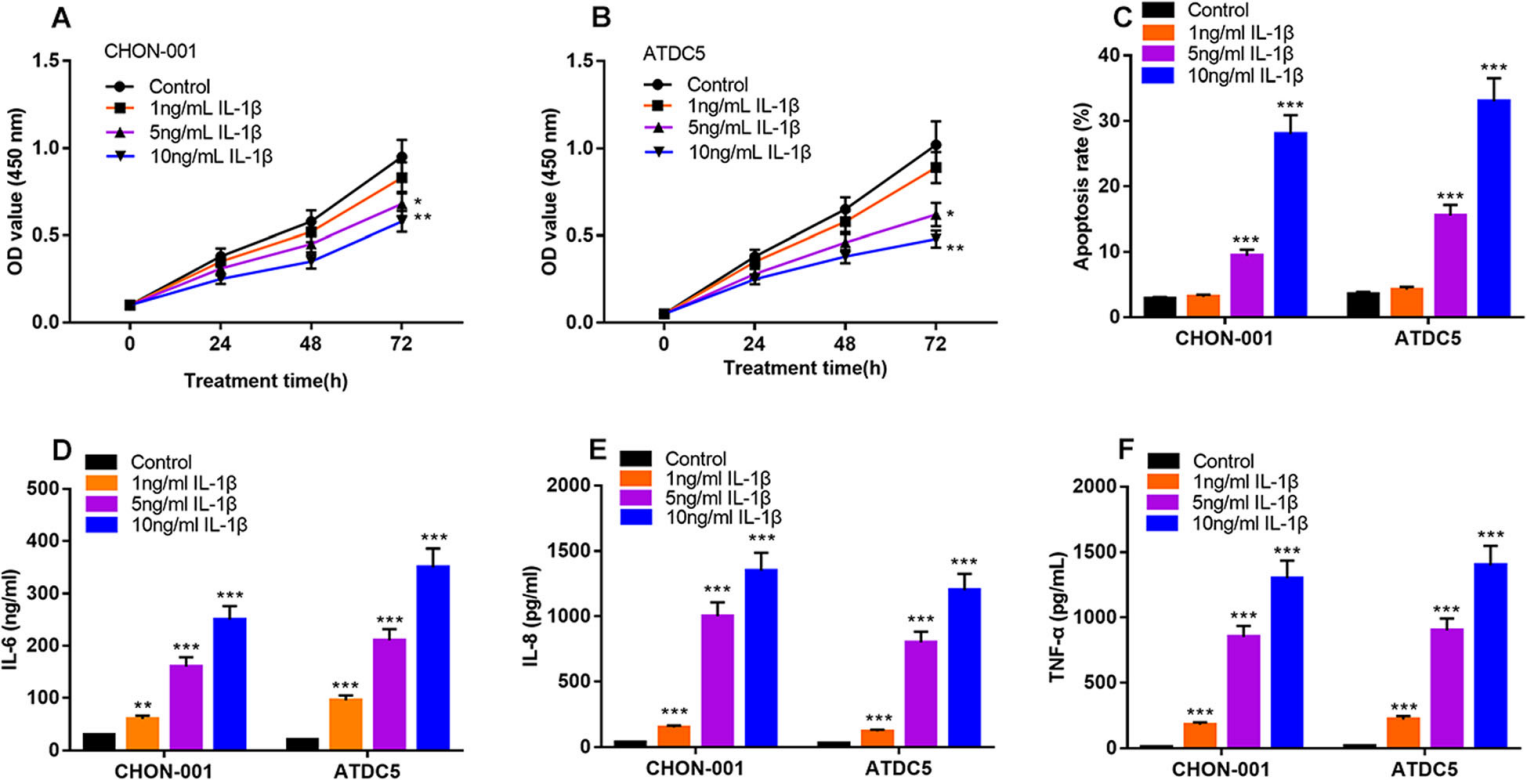
Chondrocytes were stimulated with IL-1β to construct OA model in vitro*.* **a, b** Cell viability was measured by CCK-8 method after CHON-001 and ATDC5 cells were treated with 0, 1, 5, 10 ng / mL IL-1β for 24 h. **c** CHON-001 and ATDC5 cell lines were treated with 0, 1, 5, 10 ng / mL IL-1β, and the apoptosis of CHON-001 and ATDC5 cells was detected by flow cytometry. **d-f** CHON-001 and ATDC5 cell lines were treated with 0,1,5 and 10 ng/mL IL-1β, and the contents of IL-6, IL-8 and TNF-α were detected by ELISA. *P < 0.05, **P < 0.01, ***P < 0.001 vs. control group.

First of all, CHON-001 and ATDC5 cells were treated with different concentrations of IL-1β (0, 1, 5 and 10 ng/mL). CCK-8 assay showed that the viability of the cells treated with 5 and 10 ng/mL IL-1β was significantly lower than that without IL-1β treatment, in a concentration-dependent manner (Fig. 1a & b). The results of flow cytometry analysis showed that, compared with the cells without IL-1β treatment, the apoptosis rates of the cells treated with 5 and 10 ng/mL IL-1β were significantly increased, and 10 ng/mL IL-1β induced higher apoptotic rate of chondrocytes (Fig. 1c). In addition, compared with the control group, the contents of inflammatory factors IL-6, IL-8 and TNF-α in IL-1β treatment groups were significantly increased in a concentration-dependent manner (Fig. 1d & e & f). These data suggested that IL-1β successfully induced the inflammatory injury of CHON-001 and ATDC5 cells and reduced their viability. In the subsequent experiments, the cells were stimulated with 10 ng/mL IL-1β to mimic the inflammatory environment of chondrocytes in OA pathogenesis.

### ICAR promoted the viability and inhibited the apoptosis and inflammatory response of chondrocytes induced by IL-1β

**Fig. 2:**
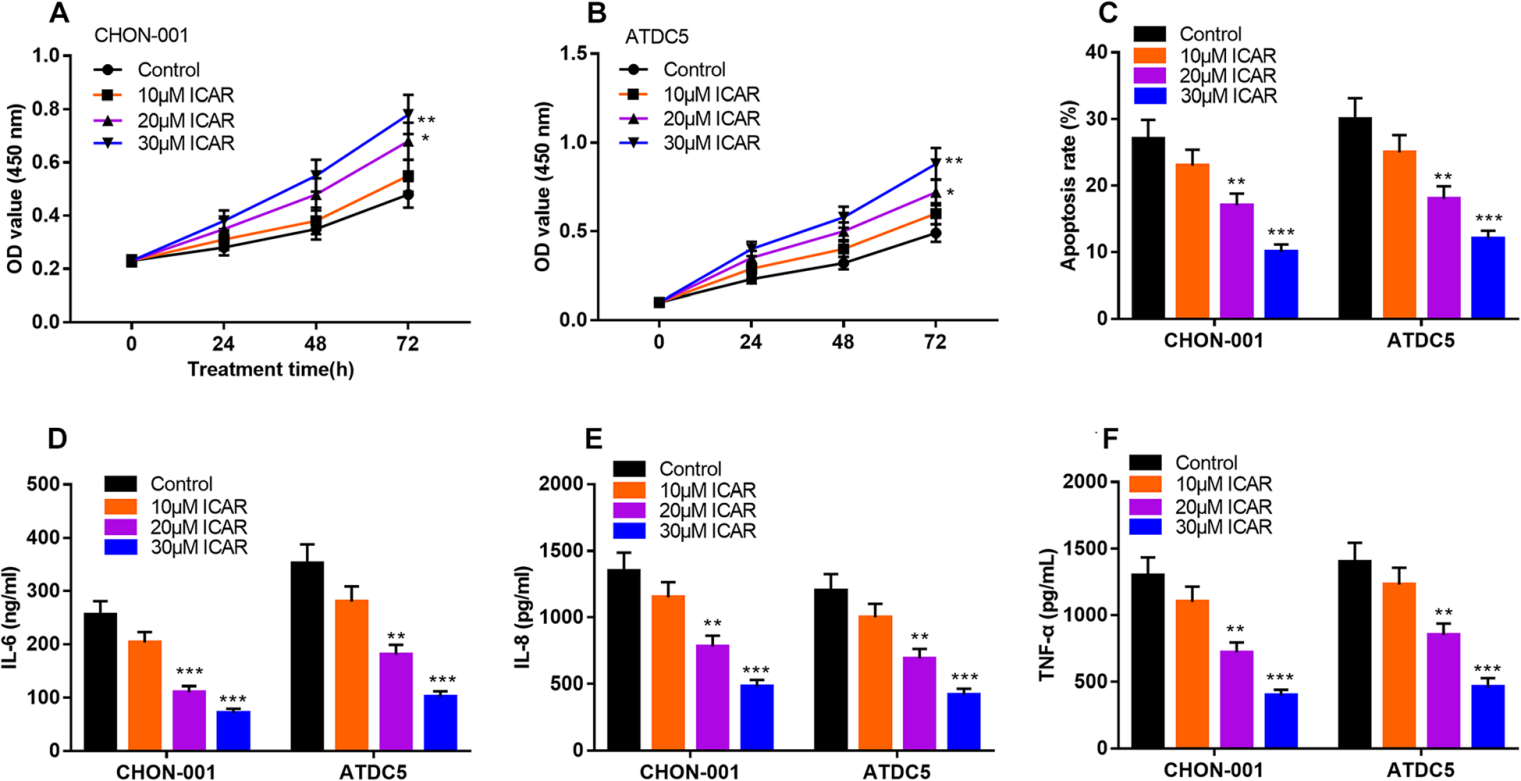
The effect of ICAR on viability, apoptosis and inflammatory damage of OA in vitro model. **a and b** The chondrocytes stimulated by IL-1β were treated with 0, 10, 20, 30 μM ICAR, respectively, and the cell viability was detected by CCK-8. **c** The chondrocytes stimulated by IL-1β were treated with 0, 10, 20, 30 μM ICAR, respectively, and the apoptosis was detected by flow cytometry. **d**-f The chondrocytes stimulated by IL-1β were treated with 0,10,20,30 μM ICAR, respectively, and the contents of IL-6, IL-8 and TNF-α were detected by ELISA. *P < 0.05, **P < 0.01, ***P < 0.001 vs. control group

After treated with IL-1β, CHON-001 and ATDC5 cells were treated with different concentrations of IACR (0, 10, 20, 30 μM), and then the viability of chondrocytes was detected by CCK-8 assay. Compared with the control group, ICAR can significantly increase the viability of chondrocytes in a concentration-dependent manner (Fig. 2a & b). Flow cytometry analysis signified that ICAR counteracted the effects of IL-1β of promoting the apoptosis of chondrocytes (Fig. 2c). Additionally, the results of ELISA showed that the contents of IL-6, IL-8 and TNF-α in ICAR treatment groups were significantly decreased, compared with the control group (Fig. 2d & e & f). These data suggested that ICAR protected chondrocytes from the inflammatory injury. Considering 30 μM ICAR had more significant effects compared with 10 μM or 20 μM ICAR, 30 μM ICAR was selected for the subsequent experiments.

### ICAR treatment specifically increased the expression of CYTOR in chondrocytes

**Fig. 3:**
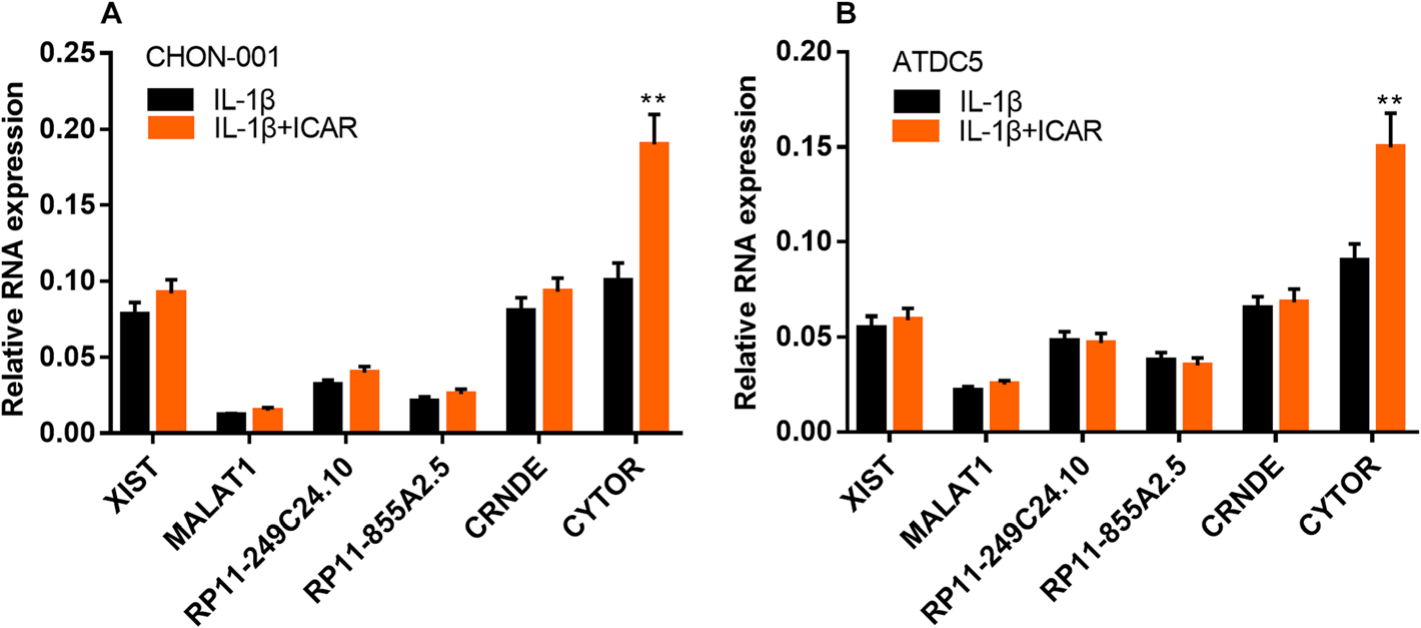
ICAR treatment up-regulated CYTOR in chondrocytes treated with IL-1β group. **b** ICAR was used to treat chondrocytes, and qRT-PCR was used to detect the expressions of 6 lncRNAs including XIST, MALAT1, RP11-249C24.10, RP11-855A2.5, CRNDE and CYTOR in chondrocytes. **P < 0.01 vs. IL-1β treatment group

Next, we performed qRT-PCR to detected several lncRNAs, which were previously reported to participate in OA pathogenesis, in CHON-001 and ATDC5 cells. These lncRNAs included XIST, MALAT1, RP11-249C24.10, RP11-855A2.5, CRNDE, and CYTOR. As shown, after CHON-001 and ATDC5 cells were treated with 30 μM ICAR, the expression of CYTOR in both cell lines was significantly up-regulated, while the other lncRNAs were not markedly affected (Fig. 3a & b). These data indicated that ICAR could probably protect chondrocytes via up-regulating CYTOR expression.

### Up-regulation of CYTOR promoted the proliferation of chondrocytes stimulated by IL-1β, and inhibited apoptosis and inflammation

**Fig. 4:**
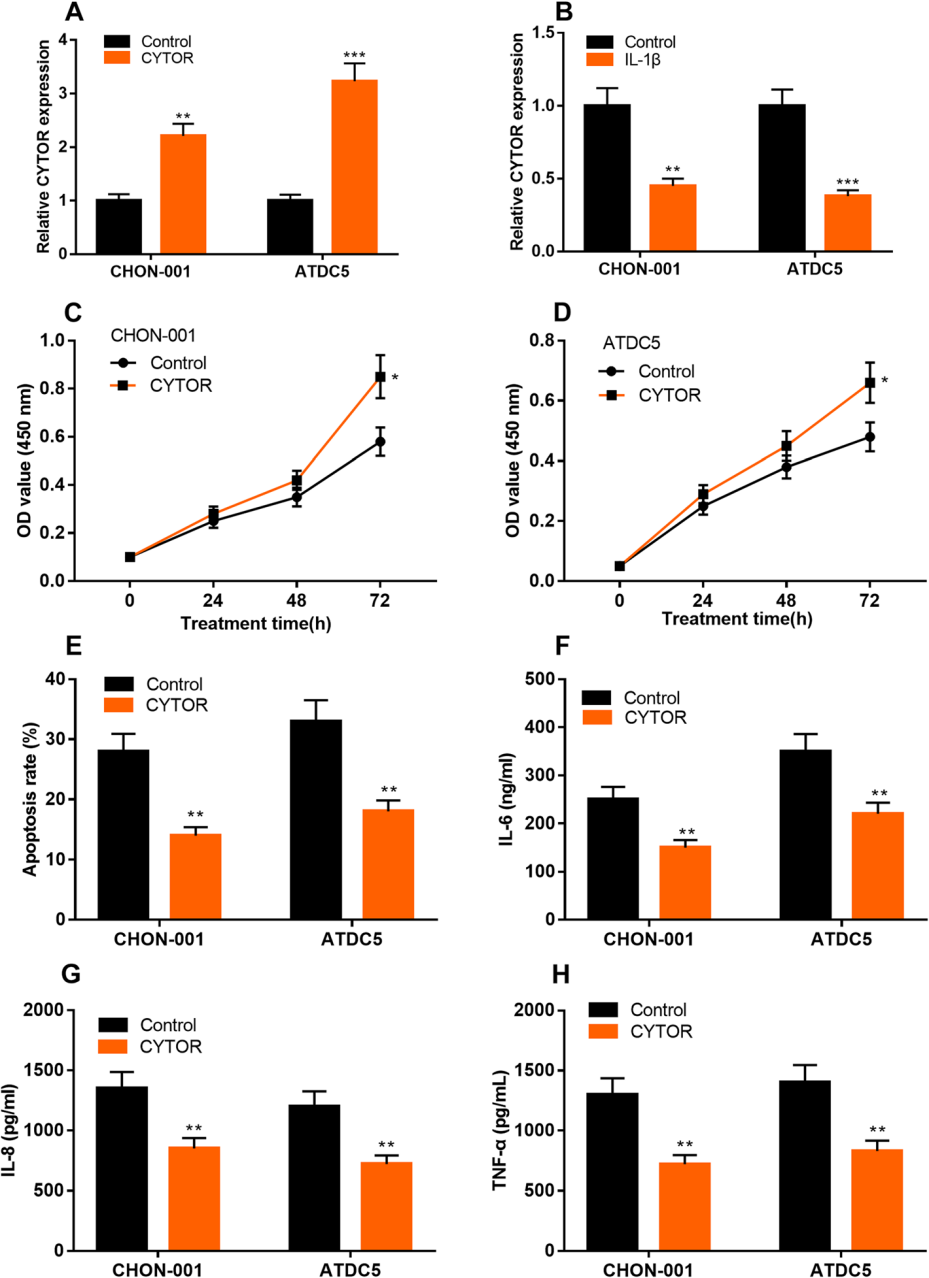
Effects of over-expressing CYTOR on the viability, apoptosis and inflammatory injury of chondrocytes. **a** CYTOR overexpression models of chondrocytes were constructed using overexpression plasmids. **b** The expression of CYTOR in chondrocytes was detected by qRT-PCR after the chondrocytes were treated with IL-1β. **c-d** The overexpression plasmid was transfected into chondrocytes, and then the cells were stimulated by IL-1β, and the cell activity was detected by CCK-8 assay. **e** After the chondrocytes were transfected with CYTOR overexpression plasmid, the cells were stimulated by IL-1β, and apoptosis was detected by flow cytometry. **f, g h** After the chondrocytes were overexpressed with CYTOR and stimulated by IL-1β, and the levels of IL-6, IL-8 and TNF-α were detected by ELISA. *P < 0.05, **P < 0.01 vs. control group (chondrocytes stimulated by IL-1β)

To further study the role of CYTOR in the progression of OA, we successfully constructed CHON-001 and ATDC5 cells with CYTOR overexpression (Fig. 4a). It was observed that IL-1β treatment significantly reduced the expression of CYTOR in both cell lines (Fig. 4b), suggesting that IL-1β might induce the injury of chondrocytes via repressing CYTOR. The results of CCK-8 assay showed that compared with the control group, the CYTOR overexpression significantly promoted the viability of chondrocytes (Fig. 4c & d). Flow cytometry analysis showed that compared with the control group, the apoptosis rate of chondrocytes was significantly reduced in CYTOR overexpression group. (Fig. 4e). In addition, compared with the control group, the levels of IL-6, IL-8 and TNF-α in the CYTOR overexpression group were significantly reduced (Fig. 4f & g & h). These results implied that CYTOR could protect chondrocytes from inflammatory injury during OA development.

### Knockdown of CYTOR reversed the protective effect of ICAR on IL-1β-stimulated chondrocytes

**Fig. 5:**
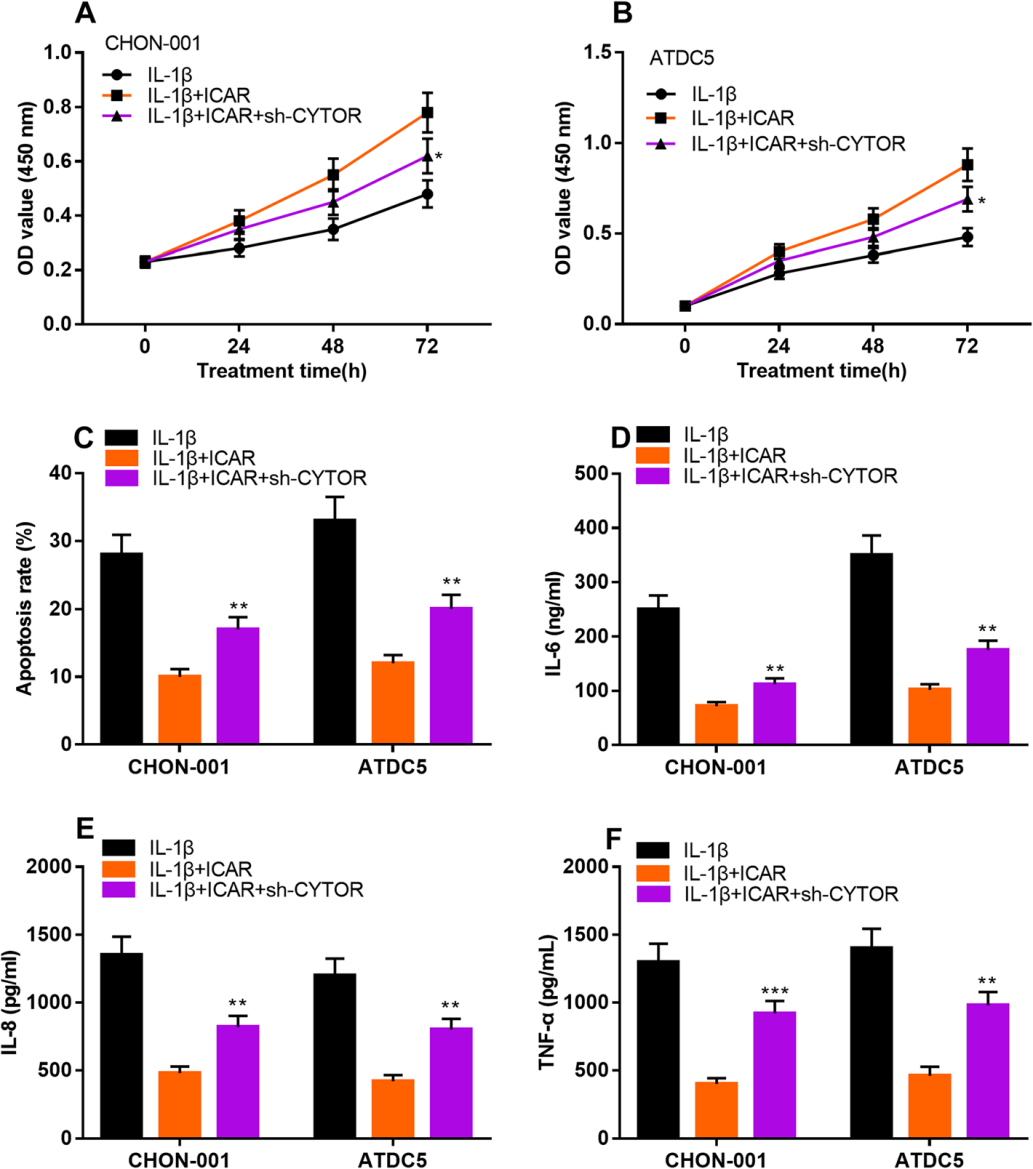
CYTOR knockdown counteracted the protective effects of ICAR on chondrocytes. **a b** The chondrocytes transfected with CYTOR shRNA were treated with ICAR, and the cell viability was detected by CCK-8. **c** The chondrocytes transfected with CYTOR shRNA were treated with ICAR, and the apoptosis was detected by flow cytometry analysis. **d**-f The chondrocytes transfected with CYTOR shRNA were treated with ICAR, and the levels of IL-6, IL-8 and TNF-α were detected by ELISA. *P < 0.05, **P < 0.01, ***P < 0.001 vs. IL-1β + ICAR group

To further analyze whether ICAR could play a role in OA through regulating CYTOR, we constructed CHON-001 and ATDC5 cells with CYTOR knockdown, and it was found that the promoting effect of ICAR on the viability of chondrocytes treated with IL-1β was counteracted by CYTOR knockdown (Fig. 5a & b). Flow cytometry assay indicated that the decrease of apoptosis rate of chondrocytes induced by ICAR was reversed by CYTOR knockdown (Fig. 5c). Consistently, the results of ELISA indicated that knockdown of CYTOR reversed the effects of ICAR treatment on reducing the levels of IL-6, IL-8, TNF-α in chondrocytes (Fig. 5d & e & f).

## Discussion

OA is a common joint degenerative disease, which is characterized by the destruction of articular cartilage, and the only cells that synthesize cartilage matrix in articular cartilage are chondrocytes. A previous study has confirmed that joint destruction in patients with OA is related to the viability of chondrocytes [10], and promoting the viability of chondrocytes may be an effective strategy for the treatment of OA. IL-1β is widely used as a stimulant to induce the inflammatory injury of chondrocytes in the studies focusing on OA pathogenesis [11]. In the present work, we observed that IL-1β treatment significantly repressed the viability, promoted the apoptosis and inflammatory response of CHON-001 and ATDC5 cells, which is consistent with the previous report.

ICAR is a kind of flavonoid glycoside extracted from Chinese herbal medicine *Epimedium*. It has various pharmacological activities, such as anti-hepatotoxicity, anti-inflammation, anti-tumor, and immune modulating function [12]. In OA, reportedly, ICAR can enhance the differentiation and mineralization of osteoblasts, inhibit bone resorption and induce osteoclast apoptosis, and ICAR can prevent bone destruction [13]. ICAR can also inhibit LPS-mediated chondrocyte injury and apoptosis by inhibiting NLRP3 and caspase-1 signaling [7]. In this study, it was validated that ICAR can promote chondrocyte viability and inhibit apoptosis and inflammatory response, suggesting that ICAR is a promising drug for OA treatment.

Although ICAR has the potential to protect chondrocytes, the regulatory mechanism still awaits more investigation. In recent years, accumulating studies indicate that lncRNA is involved in the development of OA and is related to chondrocyte viability and apoptosis. For example, lncRNA DNM3OS can adsorb miR-126 as a molecular sponge to regulate insulin-like growth factor 1 to promote chondrocyte proliferation and inhibit apoptosis [14]; lncRNA SNHG1 can alleviate IL-1β-induced injury of chondrocytes by inhibiting miR-16-5p-mediated p38 MAPK and NF-κB signaling pathways [11]. CYTOR, also known as LINC00152, is a lncRNA with a length of 828 nucleotides and expressed abnormally in many cancers, such as lung cancer, stomach cancer, colon cancer, etc. [15–18]. It is reported that CYTOR is a key lncRNA in regulating age-related articular cartilage degradation, and CYTOR expression was significantly reduced in the cartilage tissues of OA patients compared to normal samples [9]. Consistently, in the present work, we found that CYTOR is significantly down-regulated in chondrocytes treated with IL-1β. Additionally, overexpression of CYTOR can promote chondrocyte viability and inhibit its apoptosis, counteracting the effects of IL-1β. Importantly, we demonstrated that ICAR induced CYTOR expression in chondrocytes, and CYTOR knockdown reversed the protective effects on ICAR on chondrocytes. These data validated that ICAR treatment could protect the chondrocytes from inflammatory injury via regulating CYTOR.

This work has certain limitations. First of all, the downstream mechanism of ICAR and CYTOR in protecting chondrocytes is still obscure. In the present study, we found ICAR and CYTOR could regulate the levels of inflammatory cytokines (IL-6, IL-8 and TNF-α) in the OA models. Reportedly, NF-κB and p38MAPK signaling are the crucial pathways to modulate the inflammatory response and the production of cytokines in the pathogenesis of OA [19, 20]. ICAR and CYTOR may protect chondrocytes via repressing the activation of NF-κB and p38MAPK, which requires validation in the future. What’s more, in this work, only 6 lncRNAs were selected and ICAR may exert its protective function in OA development via regulating other lncRNAs. Additionally, our conclusions were based on in vitro experiments, and in vivo models should be constructed to further validate our demonstrations in the following work.

## Conclusion

In summary, this study uses an in vitro model of OA to confirm that ICAR can protect chondrocytes by up-regulating CYTOR. This finding suggested that ICAR may be a new treatment for protecting chondrocytes and alleviating the progression of OA.

## Data Availability

The data in the current study are available from the corresponding author on reasonable request.

## Abbreviations

OA: Osteoarthritis
ICAR: Icariin
LPS: Lipopolysaccharide
LncRNA: Long non-coding RNA
IL-1β: Interleukin 1β
sh-CYTOR: short hairpin RNA
qRT-PCR: quantitative real-time polymerase chain reaction
CCK-8: Cell counting kit-8
OD: Optical density
PI: Propidium iodide
ELISA: Enzyme-linked immunosorbent assay
